# Polypharmacy, drug–drug interactions and adverse drug reactions in older Chinese cancer patients: evidence from CHARLS

**DOI:** 10.3389/fphar.2025.1579023

**Published:** 2025-05-29

**Authors:** Zijun Yan, Ke-qin Fan, Ting Yu, Ning Su, Yan Zou, Liangjing Xia

**Affiliations:** Department of Pharmacy, Panzhihua Central Hospital, Panzhihua, Sichuan, China

**Keywords:** polypharmacy, drug-drug interactions, adverse drug reactions, medication safety, geriatric oncology, pharmacological interactions

## Abstract

**Objective:**

To (i) quantify the prevalence of polypharmacy and clinically significant DDIs, (ii) examine their independent and combined associations with ADRs, and (iii) explore whether depression and cognition modify these relationships among older Chinese adults with cancer among older Chinese adults diagnosed with cancer. A total of 408 participants aged ≥60 years completed both the 2011 baseline and 2013 follow-up surveys, forming the analytic cohort.

**Methods:**

This analysis used data from the China Health and Retirement Longitudinal Study (CHARLS; 2011–2013). Eligible participants were community-dwelling adults aged ≥60 years who answered “yes” to the CHARLS question “Has a doctor ever told you that you had a malignant tumour or cancer?” (variable DAOO7-4, mapped to ICD-10 C00–C97). The most frequently reported sites were lung, stomach, colorectal, liver and breast cancers, yielding an analytic cohort of 408 individuals. Polypharmacy (≥5 medications/day) was determined through face-to-face interviews, and DDIs were identified using standardized reference compendia. ADRs were confirmed by self-reports corroborated with medical records. Depression and cognition were measured using validated scales. Logistic regression models adjusted for sociodemographic and clinical factors were used to evaluate associations.

**Results:**

At baseline, 36.0% of participants reported polypharmacy, rising to 38.0% at follow-up. Clinically significant DDIs increased from 20.1% to 23.0%, while ADRs grew from 6.9% to 8.1%. In adjusted models, both polypharmacy (OR = 2.21, 95% CI = 1.14–4.30) and DDIs (OR = 3.28, 95% CI = 1.54–6.99) independently heightened ADR risk. Elevated depression scores were also linked to increased odds of ADRs, particularly among older women.

**Conclusion:**

Polypharmacy and DDIs substantially magnify the risk of ADRs in older Chinese adults with cancer, with depression further compounding vulnerability. Targeted medication management, careful DDI monitoring, and attention to psychosocial well-being may reduce preventable harms and improve outcomes in this rapidly expanding geriatric oncology population.

## 1 Introduction

Polypharmacy is a widespread public health concern worldwide, contributing to a substantial burden of medication-related morbidity and mortality that can rival or exceed the combined impact of other clinical challenges (Bandidwattanawong et al., 2023; Choi et al., 2023; Hshieh et al., 2022). Older adults with cancer, in particular, face escalating risks of adverse outcomes as multiple comorbidities, physiological decline, and complex treatment regimens converge (Ramsdale et al., 2022; Mohamed et al., 2021; Cao et al., 2023a). From 2010 onward, China has witnessed a notable increase in the incidence of cancer among its rapidly aging population, mirroring global trends in geriatric oncology. During this same period, heightened attention has been drawn to the intricate consequences of polypharmacy, including functional impairments, reduced quality of life, and increased healthcare costs (Nightingale et al., 2021; Tian et al., 2022; Albayrak et al., 2024). Many older cancer patients struggle with additional vulnerabilities, such as nutritional deficiencies and preexisting chronic diseases, which can intensify drug interactions and compound the risk of adverse reactions (Keats et al., 2021; You et al., 2023; Berger et al., 2022). These medication-related events do not merely diminish physical wellbeing but can also trigger psychological stress, social isolation, and heightened caregiver burden, fueling a cycle in which one complication leads to another and undermines adherence to necessary therapies (Whitman et al., 2021; Cao et al., 2023a; Eyigor et al., 2021).

In addition to advanced age, the most critical factors influencing medication risks in older cancer patients include multimorbidity, exposure to multiple pharmacologic agents (often prescribed by different specialists), depression, cognitive impairment, and functional limitations (Doumat et al., 2023; Pazan and Wehling, 2021; Aljeaidi and Tan, 2022). In some cases, uncoordinated prescribing amplifies the likelihood of drug-drug interactions, creating a cascade effect that can culminate in serious adverse drug reactions (Lexow et al., 2021; Lakizadeh and Babaei, 2022; Krustev et al., 2022). Existing studies suggest that patients managing both cancer treatments and various chronic conditions might be especially susceptible to potentially harmful medication overlaps (Thomsen et al., 2023; Barlow et al., 2021; Cao et al., 2022; Yang et al., 2023), yet the relationship between polypharmacy, drug-drug interactions, and adverse drug reactions remains inadequately explored, particularly among older adults in China (Yuan et al., 2023; Liu et al., 2021; Ojha and Bhargava, 2022). Although certain hospital-based data have hinted at elevated risks of polypharmacy and subsequent adverse events (Liu et al., 2023; Liu R. et al., 2024; Tang et al., 2023), there is no broad consensus on how these medication regimens intersect with clinical complexity in a nationally representative geriatric cancer population.

Recent findings also indicate a potential influence of mental health status—especially depression—on how older individuals manage their medications, further complicating the risk of adverse outcomes (Cheng and Bai, 2022; Chen et al., 2021; Yang et al., 2023). Some evidence suggests that psychological distress may impair a patient’s ability to adhere to complex treatment plans or detect early warning signs of drug-related problems (Fan et al., 2021; Tang et al., 2022; Liu X. et al., 2024). However, the current literature largely focuses on isolated aspects of medication risks, overlooking how these elements might simultaneously contribute to clinically significant drug-drug interactions and adverse drug reactions. Because depressive symptoms may disrupt medication adherence and cognitive decline can impair recognition of early drug-related problems, both constructs are integral to understanding the full pathway from polypharmacy to ADRs. We therefore assess depression and cognition as potential effect modifiers, while retaining a concise title centred on the primary pharmacological exposures. The interplay between cognitive decline, emotional wellbeing, and increasing numbers of prescriptions could form a matrix of risks that disproportionately affects older cancer patients.

In the context of China’s deepening aging trend and the rising prevalence of cancer in later life, this study aims to (1) measure the prevalence of polypharmacy and DDIs, (2) evaluate their separate and joint effects on ADRs, and (3) test whether depression and global cognition moderate these associations in a nationally representative geriatric-oncology cohort. By drawing on a nationally representative longitudinal dataset, this research seeks to provide targeted insights for health practitioners and policymakers, ultimately helping to develop strategies that mitigate the detrimental impact of overprescribing, reduce preventable medication harms, and improve the overall wellbeing of geriatric oncology patients in China.

## 2 Method

### 2.1 Participants

**FIGURE 1:**
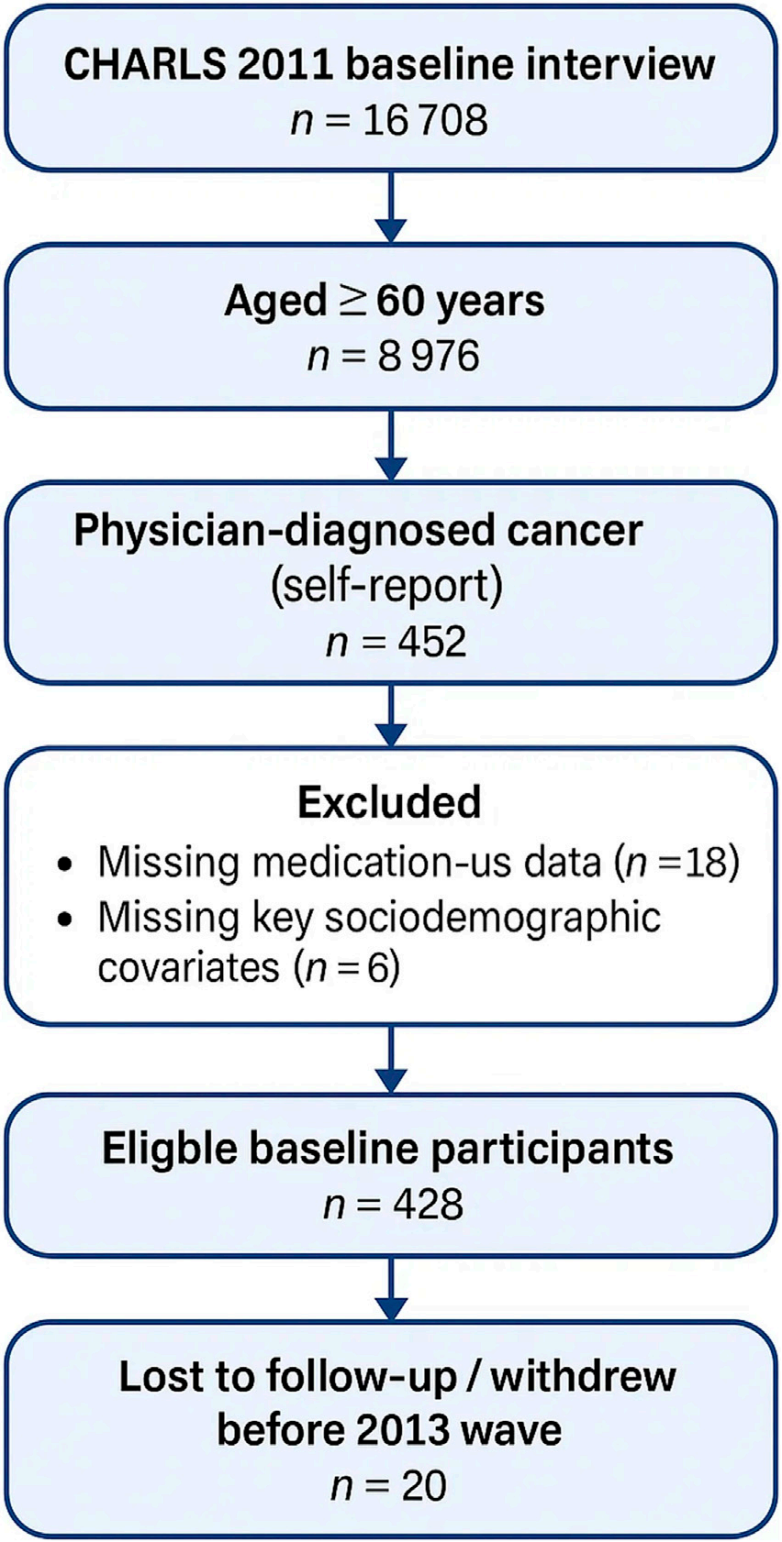
Participant-selection flowchart.

Data were obtained from the China Health and Retirement Longitudinal Study (CHARLS), a multistage, stratified survey that has tracked a nationally representative cohort of Chinese adults aged ≥45 years since 2011, with biennial follow-ups. For the present analysis we first isolated respondents aged ≥60 years at the 2011 baseline and then applied CHARLS variable DAOO7-4 (“Has a doctor ever told you that you had a malignant tumour or cancer?”) to operationalise physician-diagnosed cancer. This item captures self-reported physician confirmation of any malignant neoplasm and is harmonised to ICD-10 codes C00–C97 in the public dataset. Participants missing medication data, key covariates or 2013 follow-up interviews were excluded, leaving 408 eligible individuals. Ethical clearance for CHARLS was granted by the Peking University Institutional Review Board (IRB00001052-11015), and all respondents provided written informed consent. Figure 1 summarises the participant-selection flowchart, including reasons for exclusion at every stage. All individuals provided written informed consent, the Peking University Ethics Committee has approved all relevant research for CHARLS, with approval number IRB00001052-11015.

### 2.2 Polypharmacy, drug-drug interactions, and adverse drug reactions

Information on prescription and over-the-counter medications used by participants in the month prior to the interview was collected through structured, face-to-face questionnaires administered by trained CHARLS interviewers. Polypharmacy was defined as the use of five or more distinct medications per day. This threshold follows the 2023 Chinese Geriatric Pharmacotherapy Guidelines and is consistent with widely cited international criteria for inappropriate polypharmacy. Drug-drug interactions (DDIs) were identified by cross-referencing each participant’s self-reported medication list against recognized pharmacological references commonly utilized in Chinese hospital settings and international drug interaction databases (Ojha and Bhargava, 2022; Iwatsubo, 2020; Marengoni and Onder, 2015; Sudsakorn et al., 2020; Floor-Schreudering et al., 2014). Adverse drug reactions (ADRs) were ascertained by asking participants if they had experienced any new or unexpected health problems they believed to be related to medication use. ADR identification depended chiefly on participants’ self-reports of medication-related events.

### 2.3 Depression and cognition

Depression was assessed using the 10-item Center for Epidemiological Studies Depression Scale (CES-D-10) (Chen and Mui, 2014). Participants were asked to rate the frequency of depressive symptoms experienced in the past week on a four-point Likert scale. Total scores ranged from 0 to 30, with higher scores indicating more severe depressive symptoms.

Cognitive function was measured by a brief battery assessing memory, orientation, and basic numeracy, adapted from the CHARLS Harmonized Cognitive Assessment Protocol (HCAP) (Langa et al., 2020). Respondents were required to recall items from a word list, perform serial subtractions, and correctly identify the current year, season, and day of the week. Scores from each section were summed to generate a global cognition score, with higher scores reflecting better cognitive function.

### 2.4 Other covariates

Sociodemographic variables included age, sex, marital status (married with spouse present vs. other), and highest educational attainment (illiterate, primary school, middle school, or high school/vocational school and above). Lifestyle variables included smoking status (current or former smoker vs. never smoker) and alcohol intake (none, less than once per month, or once a month and above). Medical history and comorbidities, such as hypertension, diabetes, stroke, and heart disease, were obtained from self-reports. Additionally, participants were asked to report any prior history of medication-related problems.

### 2.5 Statistical analysis

Continuous variables were presented as mean ± standard deviation (SD) if normally distributed or as median (interquartile range) if not normally distributed. Categorical variables were summarized using frequency (N) and proportion (%). Chi-square tests and Mann-Whitney U-tests were used where appropriate to compare the distributions of participant characteristics according to the presence or absence of drug-drug interactions and ADRs at follow-up. To investigate the associations among polypharmacy, drug-drug interactions, and ADRs, logistic regression analyses were performed, controlling for potential confounders including age, sex, marital status, education, smoking status, alcohol use, depression, cognition, and the number of comorbid chronic conditions. Both unadjusted and adjusted odds ratios (ORs) with 95% confidence intervals (CIs) were calculated. All analyses were conducted using R version 4.2.1, and p-values <0.05 were considered statistically significant.

## 3 Result

### 3.1 Participants’ characteristics

A total of 408 older adults (mean age 69.1 ± 6.3 years) with physician-diagnosed cancer at baseline were followed up from 2011 to 2013. Among them, 222 (54.4%) were male, and 186 (45.6%) were female. The five most commonly reported primary cancer sites were lung (14.0%), stomach (12.3%), colorectal (10.8%), liver (8.6%), and breast (7.6%; 16.1% among women), with the remainder comprising oesophageal, prostate, nasopharyngeal, bladder and other solid or haematological malignancies. Table 1 shows the sociodemographic characteristics, number of comorbidities, and baseline clinical information of the participants. More than half (55.9%) were aged 60–69, while 31.4% and 12.7% were in the 70–79 and ≥80 age brackets, respectively. The median number of chronic diseases was 1 (interquartile range: 1–2). Approximately 36.0% (n = 147) had polypharmacy, defined as the use of five or more medications per day. The mean depression (CES-D-10) score at baseline was 8.2 ± 4.1, whereas the mean cognition score was 12.5 ± 4.0.

**TABLE 1:** Baseline characteristics of the study participants (N = 408).

Characteristic	Total (N = 408)	Male (N = 222)	Female (N = 186)
Age (years), mean ± SD	69.1 ± 6.3	69.5 ± 6.2	68.7 ± 6.4
Age group, n (%)			
60–69	228 (55.9)	121 (54.5)	107 (57.5)
70–79	128 (31.4)	72 (32.4)	56 (30.1)
≥80	52 (12.7)	29 (13.1)	23 (12.4)
Marital status, n (%)			
Married	309 (75.7)	175 (78.8)	134 (72.0)
Separated/Divorced/Widowed/Other	99 (24.3)	47 (21.2)	52 (28.0)
Education, n (%)			
Illiterate/No formal education	103 (25.2)	38 (17.1)	65 (34.9)
Primary school	196 (48.0)	118 (53.2)	78 (41.9)
Middle school	72 (17.6)	38 (17.1)	34 (18.3)
High school and above	37 (9.1)	28 (12.6)	9 (4.8)
Number of chronic diseases, n (%)			
0	42 (10.3)	19 (8.6)	23 (12.4)
1 or 2	199 (48.8)	112 (50.5)	87 (46.8)
≥3	167 (40.9)	91 (41.0)	76 (40.9)
Polypharmacy, n (%)			
Yes (≥5 medications)	147 (36.0)	75 (33.8)	72 (38.7)
No (<5 medications)	261 (64.0)	147 (66.2)	114 (61.3)
Depression (CES-D-10), mean ± SD	8.2 ± 4.1	7.6 ± 3.9	8.7 ± 4.3
Cognition score, mean ± SD	12.5 ± 4.0	12.9 ± 4.3	12.1 ± 3.7

### 3.2 Prevalence of polypharmacy, drug-drug interactions, and adverse drug reactions

Table 2 presents the distribution of polypharmacy, drug-drug interactions (DDIs), and adverse drug reactions (ADRs) at baseline (2011) and follow-up (2013). Overall, 147 (36.0%) participants reported polypharmacy at baseline, which slightly increased to 155 (38.0%) at follow-up. Of those with polypharmacy at baseline, 58 (39.5%) experienced at least one clinically significant DDI, compared to 24 (9.2%) among those without polypharmacy (p < 0.001). The total prevalence of DDIs rose from 82 (20.1%) at baseline to 94 (23.0%) at follow-up. Self-reported ADRs, corroborated whenever possible with medical notes, were observed in 28 (6.9%) participants at baseline and 33 (8.1%) at follow-up. At follow-up, the ten most frequent clinically significant drug–drug interaction (DDI) pairs were: aspirin + clopidogrel (14.9% of all DDIs), warfarin + fluoroquinolones (10.6%), omeprazole + clopidogrel (9.6%), metformin + iodinated contrast agents (8.5%), cisplatin + furosemide (7.4%), vincristine + azoles (6.4%), warfarin + carbamazepine (5.3%), methotrexate + NSAIDs (4.3%), dexamethasone + insulin/other antidiabetics (4.3%), and tramadol + SSRIs/SNRIs (3.2%). The corresponding adverse drug reactions (ADRs) most frequently documented were gastrointestinal bleeding (18.2% of all ADRs), neutropenia (15.2%), renal impairment (12.1%), transaminase elevation (12.1%), severe nausea/vomiting (9.1%), hypoglycaemia (9.1%), hypersensitivity rash (9.1%), ototoxicity (6.1%), serotonin syndrome (3.0%), and thrombo-embolism (3.0%). Overall, 28/33 ADR cases at follow-up (84.8%) occurred in participants who also harboured at least one of the above DDIs, underscoring the clinical relevance of these interaction clusters. The specific details are shown in Supplementary Tables S5, S6 in the supplementary document.

**TABLE 2:** Distribution of polypharmacy, DDIs, and ADRs at baseline and follow-up (N = 408).

Variable	2011 Baseline, n (%)	2013 Follow-up, n (%)
Polypharmacy (≥5 medications)	147 (36.0)	155 (38.0)
DDI prevalence (overall)	82 (20.1)	94 (23.0)
Among polypharmacy group	58 (39.5)	63 (40.6)
Among non-polypharmacy group	24 (9.2)	31 (11.9)
ADR prevalence (overall)	28 (6.9)	33 (8.1)
Among participants with DDI	23 (28.0)	28 (29.8)
Among participants without DDI	5 (1.8)	5 (1.9)

### 3.3 Comparison of participant characteristics by drug-drug interactions and ADRs

Table 3 compares demographic and clinical characteristics by the presence or absence of DDIs and ADRs at follow-up. Participants with DDIs (n = 94) had a significantly higher mean number of medications (5.6 ± 1.8) than those without DDIs (3.2 ± 1.3), and they were more likely to have three or more chronic conditions (53.2% vs. 37.4%, p = 0.008). The depression score was higher in those with DDIs (9.1 ± 4.2) compared to those without (7.9 ± 3.9, p = 0.020). Among participants with ADRs (n = 33), polypharmacy was present in 24 (72.7%), and 28 (84.8%) had at least one DDI.

**TABLE 3:** Comparison of characteristics by presence of DDIs and ADRs at follow-up (N = 408).

Characteristic	DDI (n = 94)	No DDI (n = 314)	p-value (DDI vs. No DDI)	ADR (n = 33)	No ADR (n = 375)	p-value (ADR vs. No ADR)
Mean age, years (±SD)	69.7 ± 6.4	68.9 ± 6.2	0.253	70.0 ± 6.6	69.0 ± 6.2	0.287
Polypharmacy, n (%)	63 (67.0)	92 (29.3)	<0.001	24 (72.7)	131 (34.9)	<0.001
Mean number of medications	5.6 ± 1.8	3.2 ± 1.3	0.015	6.2 ± 2.3	3.1 ± 1.5	0.185
≥3 chronic conditions, n (%)	50 (53.2)	117 (37.3)	0.008	23 (69.7)	144 (38.4)	0.001
Depression score (±SD)	9.1 ± 4.2	7.9 ± 3.9	0.02	9.8 ± 4.6	8.0 ± 4.0	0.012
Cognition score (±SD)	12.0 ± 4.1	12.6 ± 3.9	0.164	11.6 ± 4.3	12.5 ± 4.0	0.213
ADR, n (%)	28 (29.8)	5 (1.6)	<0.001	—	—	—

### 3.4 Association of polypharmacy and DDIs with ADRs

Logistic regression analyses were conducted to examine the associations among polypharmacy, DDIs, and ADRs while adjusting for potential confounders, including age, sex, marital status, education, smoking status, alcohol use, depression, cognition, and the number of chronic conditions (Table 4). In the fully adjusted model, participants with polypharmacy had 2.21 times higher odds of experiencing an ADR compared to those without polypharmacy (95% CI: 1.14–4.30). The presence of a clinically significant DDI was also independently associated with ADRs (OR = 3.28; 95% CI: 1.54–6.99). Additionally, higher depression scores were associated with increased odds of having ADRs (OR = 1.05; 95% CI: 1.01–1.10).

**TABLE 4:** Logistic regression for ADRs associated with polypharmacy and DDIs (N = 408).

Variable	Crude OR (95% CI)	p-value	Adjusted OR (95% CI)	p-value
Polypharmacy (Yes vs. No)	2.53 (1.33–4.80)	0.005	2.21 (1.14–4.30)	0.019
DDI (Yes vs. No)	4.09 (2.11–7.93)	<0.001	3.28 (1.54–6.99)	0.002
Depression score (per 1-point)	1.04 (1.00–1.08)	0.049	1.05 (1.01–1.10)	0.015
Cognition score (per 1-point)	0.98 (0.94–1.02)	0.349	0.97 (0.93–1.02)	0.278
Age (years)	1.01 (0.97–1.05)	0.663	1.02 (0.97–1.06)	0.363
Female (vs. Male)	1.16 (0.63–2.12)	0.631	1.32 (0.69–2.53)	0.402
≥3 chronic conditions (vs. <3)	1.57 (0.87–2.82)	0.134	1.39 (0.76–2.54)	0.286

### 3.5 Subgroup analysis

We performed a subgroup analysis stratified by sex to explore whether the associations of polypharmacy and DDIs with ADRs differ between older men and women. As shown in Table 5, the adjusted odds ratios for polypharmacy and DDIs remained significant in both subgroups, though the magnitude varied slightly. Among male participants, polypharmacy was associated with 2.18 times higher odds of experiencing an ADR (95% CI: 1.00–4.79), and clinically significant DDIs increased the ADR risk by 3.11 times (95% CI: 1.27–7.63). In females, the presence of polypharmacy also demonstrated a consistent association (adjusted OR = 2.29; 95% CI: 1.07–4.90), and DDIs elevated the ADR risk more than threefold (adjusted OR = 3.40; 95% CI: 1.44–8.06). Additionally, higher depression scores were notably linked with elevated odds of ADR in women (adjusted OR = 1.07; 95% CI: 1.02–1.12), whereas the confidence intervals in men slightly overlapped 1, suggesting a more modest effect in the male subgroup. These findings underscore the importance of tailored medication management strategies that consider sex-specific risk factors in geriatric cancer populations.

**TABLE 5:** Subgroup analysis of associations between polypharmacy, DDIs, and ADRs by sex.

Variable	Men (N = 222) Crude OR (95% CI)	p-value	Men adjusted OR (95% CI)	p-value	Women (N = 186) Crude OR (95% CI)	p-value	Women adjusted OR (95% CI)	p-value
Polypharmacy (Yes vs. No)	2.60 (1.10–6.20)	0.028	2.18 (1.00–4.79)	0.049	2.45 (1.12–5.35)	0.024	2.29 (1.07–4.90)	0.032
DDI (Yes vs. No)	3.82 (1.70–8.57)	0.001	3.11 (1.27–7.63)	0.013	4.21 (1.83–9.60)	<0.001	3.40 (1.44–8.06)	0.005
Depression score (per 1-point)	1.02 (0.94–1.10)	0.576	1.03 (0.95–1.11)	0.464	1.06 (1.01–1.11)	0.023	1.07 (1.02–1.12)	0.015

All adjusted models control for age, marital status, education, smoking status, alcohol use, cognition, and number of chronic conditions. Abbreviations: OR, odds ratio; CI, confidence interval; DDI, drug-drug interaction; ADR, adverse drug reaction.

## 4 Discussion

In some countries, older cancer patients face a pronounced risk of medication-related complications, similar to the patterns seen in our study of geriatric oncology patients drawn from the CHARLS cohort. Building upon the existing literature suggesting that multimorbidity and complex treatment regimens often predispose older adults to poor health outcomes (Lee et al., 2022), our findings highlight the prevalence of polypharmacy and its notable associations with drug-drug interactions (DDIs) and adverse drug reactions (ADRs). From 2011 to 2013, the proportion of individuals taking five or more medications rose, paralleling an increase in clinically significant DDIs and ADRs. The adjusted odds ratios (2.21 for polypharmacy and 3.28 for DDIs) equate to roughly a two- to three-fold jump in ADR probability—an effect size large enough to merit routine, proactive medication reviews in oncology practice. These results underscore the vulnerability of older Chinese adults with cancer to medication-related events, given their higher likelihood of chronic comorbidities and potentially heightened sensitivity to pharmaceutical interventions.

The present study also reveals several noteworthy nuances. Echoing evidence that sex influences pharmacokinetics, prescribing patterns, and health-seeking behaviour, our subgroup analyses showed raised ADR odds in both sexes, with slightly higher estimates in women—possibly reflecting differences in body composition, metabolic-enzyme activity, and social factors such as fragmented care and lower access to deprescribing services. For older men, polypharmacy appeared to confer a slightly lower odds ratio for ADRs than in women, but it nonetheless underscores the importance of carefully reviewing and coordinating multiple prescriptions (Jungo et al., 2021; Cao et al., 2023b; Tian et al., 2021). Meanwhile, older women, who showed a significant association between higher depression scores and ADRs, may require particular attention to psychosocial factors. This pattern is in line with evidence that women can be more susceptible to social and emotional stressors, which may amplify medication-related issues and compound underlying health conditions. Such findings not only point to the need for patient-centered medication management strategies but also highlight the importance of psychological support in reducing adverse events.

In addition, our results underscore the interplay of polypharmacy with complex regimens for cancer and other chronic diseases. These older adults often juggle a constellation of drugs, which can heighten the risk of synergistic or antagonistic interactions, ultimately tipping the balance toward unfavorable outcomes. The role of depression further emerges as a salient element. Consistent with previous work showing that emotional well-being can influence how older people manage and experience treatment (Reynolds et al., 2022; Zazzara et al., 2021; Wampold and Flückiger, 2023), our study indicates that higher depressive symptom scores are associated with an increased likelihood of ADRs. One possible explanation is that depressive symptoms may lead to alterations in medication adherence, dietary habits, and physiological processes, each contributing to a heightened risk of adverse events. Depression-related dysregulation of the hypothalamic–pituitary–adrenal axis and pro-inflammatory signalling can also modify cytochrome P450 activity, altering drug metabolism and potentiating dose-dependent toxicities. These findings emphasize the multifaceted challenges of medication management in geriatric oncology. Because ADR prevalence was below 10%, the reported odds ratios closely approximate relative risks, minimising the risk of misinterpretation. Monitoring and minimizing polypharmacy, regularly screening for clinically significant DDIs, and addressing depressive symptoms may help reduce the burden of ADRs in this population.

Our study has several advantages. Firstly, we leveraged data from CHARLS, a nationally representative longitudinal survey, thereby enhancing the generalizability of our findings to a broad population of older Chinese adults with cancer. However, our study also has notable limitations. First CHARLS participants are community-dwelling older Chinese adults; differences in health-system context, formularies, and supportive-care models mean caution is needed when extrapolating these estimates to hospital-based cohorts or non-Chinese settings. Second, ADRs were identified through participant or family recall corroborated, where possible, by medical charts; such retrospective reporting can miss short-lived or subclinical reactions and may differentially misclassify events among cognitively impaired respondents, underscoring the need for prospective, standardised pharmacovigilance in future work. We didn't have longitudinal measures of changes in depression or cognitive function over time, factors that may fluctuate and, in turn, alter medication adherence and vulnerability to ADRs. Although exposure preceded ADR assessment in our timeline, early ADRs could still have influenced subsequent prescribing, a potential reverse-causality pathway warranting exploration in longer follow-up waves. Future research in larger and more diverse cohorts should incorporate more detailed measures of drug utilization and integrate advanced analytic techniques to explore synergistic medication interactions.

## 5 Conclusion

This study highlights the intricate associations of polypharmacy and clinically significant drug-drug interactions (DDIs) with adverse drug reactions (ADRs) in older Chinese adults diagnosed with cancer, noting sex- and depression-specific differences in these relationships. While polypharmacy and the presence of DDIs were closely linked to an increased likelihood of experiencing ADRs, the magnitude of these associations appeared more pronounced among individuals who reported higher depressive symptoms. Notably, older women demonstrated heightened vulnerability when both polypharmacy and depressive symptoms coexisted, whereas older men showed elevated ADR risk primarily in the context of significant DDIs.These findings suggest that individualized medication management strategies, especially those taking into account sex differences and mental health—may prove beneficial in reducing ADRs in geriatric oncology populations. Future studies should also consider integrating more refined measures of drug utilization, frequent screenings for depression, and longitudinal assessments of changes in pharmacotherapy to better capture the complexities of medication risk in this population.

## Data Availability

The original contributions presented in the study are included in the article/Supplementary Material, further inquiries can be directed to the corresponding author.
