# Supplementary material for: Polypharmacy, drug–drug interactions and adverse drug reactions in older Chinese cancer patients: evidence from CHARLS
Source: Front Pharmacol. 2025 May 29;16:1579023. doi: 10.3389/fphar.2025.1579023 (PMC12158949; doi:10.3389/fphar.2025.1579023)
Supplement: Supplementary file 1 [file Supplementaryfile1.docx]

**Table S1. Classification of Clinically Significant Drug-Drug Interactions (DDIs) Identified at Baseline and Follow-up**

| **Medication Class Pairs** | **Example Agents** | **Participants with the Interaction (Baseline)*** | **Participants with the Interaction (Follow-up)*** |
| --- | --- | --- | --- |
| Cardiovascular drugs + NSAIDs | Beta-blocker (metoprolol) + Ibuprofen | 25 | 28 |
| Anticoagulants + Antiplatelets | Warfarin + Aspirin | 19 | 22 |
| Opioids + Benzodiazepines | Tramadol + Diazepam | 14 | 16 |
| ACE Inhibitors + Potassium-sparing diuretics | Enalapril + Spironolactone | 9 | 10 |
| SSRIs + NSAIDs | Sertraline + Ibuprofen | 23 | 26 |
| **Total Reported Interactions** | – | **≈90** | **≈102** |

**Table S2. Prevalence of Polypharmacy by Age Group and Sex at Baseline (2011) and Follow-up (2013)**

| **Age Group (years)** | **Total Participants** | **Polypharmacy at Baseline (n, %)** | **Polypharmacy at Follow-up (n, %)** |
| --- | --- | --- | --- |
| 60–69 | 228 | 68 (29.8) | 71 (31.1) |
| 70–79 | 128 | 52 (40.6) | 55 (43.0) |
| ≥80 | 52 | 27 (51.9) | 29 (55.8) |
| **Total** | **408** | **147 (36.0)** | **155 (38.0)** |

| **Sex** | **Total Participants** | **Polypharmacy at Baseline (n, %)** | **Polypharmacy at Follow-up (n, %)** |
| --- | --- | --- | --- |
| Male | 222 | 75 (33.8) | 80 (36.0) |
| Female | 186 | 72 (38.7) | 75 (40.3) |
| **Total** | **408** | **147 (36.0)** | **155 (38.0)** |

**Table S3. Variance Inflation Factors (VIF) in the Final Logistic Regression Models**

| **Variable** | **Overall Model VIF** | **Male Subgroup VIF** | **Female Subgroup VIF** | **df*** |
| --- | --- | --- | --- | --- |
| Age (continuous) | 1.24 | 1.2 | 1.33 | 1 |
| Sex (male vs. female) | 1.82 | – | – | 1 |
| Marital Status | 1.11 | 1.08 | 1.15 | 1 |
| Education (categorical) | 1.27 | 1.19 | 1.31 | 3 |
| Smoking Status | 1.18 | 1.06 | 1.04 | 1 |
| Alcohol Use | 1.14 | 1.1 | 1.09 | 2 |
| ≥3 Chronic Conditions | 1.13 | 1.15 | 1.14 | 1 |
| Depression Score (CES-D-10) | 1.29 | 1.25 | 1.32 | 1 |
| Cognition Score | 1.12 | 1.1 | 1.11 | 1 |
| Polypharmacy (≥5 meds) | 1.22 | 1.19 | 1.25 | 1 |
| Drug-Drug Interaction (yes) | 1.36 | 1.31 | 1.38 | 1 |

**Table S4. Hosmer–Lemeshow Goodness-of-Fit Tests for Logistic Regression Models**

| **Model** | **Chi-squared** | **df** | ***p***-value |
| --- | --- | --- | --- |
| Overall Model | 6.92 | 8 | 0.545 |
| Male Subgroup Model | 7.18 | 8 | 0.517 |
| Female Subgroup Model | 5.44 | 8 | 0.711 |

**Table S5. All 25 clinically significant drug–drug interaction (DDI) pairs observed at the 2013 follow-up, ranked by frequency.**

| **Rank** | **Drug 1** | **Drug 2** | **Principal pharmacological mechanism** | **Frequent clinical consequence(s)** | **n (%) of all DDIs*** |
| --- | --- | --- | --- | --- | --- |
| 1 | Aspirin | Clopidogrel | Additive platelet inhibition | Major GI / intracranial bleeding | 14 (14.29%) |
| 2 | Warfarin | Ciprofloxacin / Levofloxacin | CYP1A2 / CYP3A4 inhibition ± vitamin-K gut-flora loss | ↑ INR, bleeding | 10 (10.2%) |
| 3 | Omeprazole / Esomeprazole | Clopidogrel | CYP2C19 inhibition | ↓ Clopidogrel activation → stent thrombosis | 9 (9.18%) |
| 4 | Metformin | Iodinated contrast media | Contrast-induced renal decline | Lactic acidosis | 8 (8.16%) |
| 5 | Cisplatin | Furosemide | Synergistic ototoxicity & nephrotoxicity | Sensorineural hearing loss, AKI | 7 (7.14%) |
| 6 | Vincristine | Itraconazole / Fluconazole | CYP3A4 inhibition | Vincristine-induced neuropathy | 6 (6.12%) |
| 7 | Warfarin | Carbamazepine / Phenytoin | CYP induction | ↓ INR → thrombosis | 5 (5.1%) |
| 8 | Methotrexate | NSAIDs (Ibuprofen, Diclofenac) | ↓ MTX renal clearance | Pancytopenia, mucositis | 4 (4.08%) |
| 9 | Dexamethasone | Insulin / Sulfonylureas | Hyperglycaemic effect | Poor glycaemic control, hypoglycaemia swings | 4 (4.08%) |
| 10 | Tramadol | SSRIs / SNRIs | Excess serotonergic activity | Serotonin syndrome, seizures | 3 (3.06%) |
| 11 | Warfarin | Amiodarone | CYP2C9 inhibition | ↑ INR, haemorrhage | 3 (3.06%) |
| 12 | Capecitabine | Proton-pump inhibitors | pH-dependent absorption ↓ | Sub-therapeutic 5-FU exposure | 3 (3.06%) |
| 13 | Tamoxifen | Paroxetine / Fluoxetine | CYP2D6 inhibition | ↓ Endoxifen → cancer relapse | 3 (3.06%) |
| 14 | Digoxin | Verapamil | P-gp inhibition | Digoxin toxicity (bradycardia) | 2 (2.04%) |
| 15 | Clopidogrel | Voriconazole | CYP inhibition | ↑ Bleeding risk | 2 (2.04%) |
| 16 | Cisplatin | Aminoglycosides | Additive nephro- & ototoxicity | AKI, deafness | 2 (2.04%) |
| 17 | Paclitaxel | Ketoconazole | CYP3A4 inhibition | Severe neutropenia | 2 (2.04%) |
| 18 | Warfarin | Fluconazole | CYP2C9 inhibition | ↑ INR, bleeding | 2 (2.04%) |
| 19 | Allopurinol | Azathioprine | Xanthine-oxidase blockade | Profound myelosuppression | 2 (2.04%) |
| 20 | Methotrexate | Trimethoprim–Sulfa | Dual folate antagonism | Pancytopenia, AKI | 2 (2.04%) |
| 21 | Lisinopril / Other ACE-I | Spironolactone | Additive potassium retention | Hyperkalaemia, arrhythmia | 1 (1.02%) |
| 22 | Aspirin | SSRIs (Sertraline, Paroxetine) | Platelet inhibition + ↑ gastric acid | Upper-GI bleeding | 1 (1.02%) |
| 23 | Digoxin | Clarithromycin / Erythromycin | P-gp & CYP3A4 inhibition | Digoxin toxicity (arrhythmia) | 1 (1.02%) |
| 24 | β-Blocker (Metoprolol) | Verapamil / Diltiazem | Additive AV-node suppression | Severe bradycardia, heart block | 1 (1.02%) |
| 25 | Cyclophosphamide | Doxorubicin | Cumulative cardiotoxicity | Congestive heart failure | 1 (1.02%) |

**Table S6. Spectrum and frequency of adverse drug reactions (ADRs) documented at follow-up (N = 33 ADR cases).**

| **Rank** | **System-Organ Class / Preferred term** | **Common culprit drug(s)†** | **n** | **% of ADRs** |
| --- | --- | --- | --- | --- |
| 1 | Gastro-intestinal bleeding | Aspirin ± clopidogrel, warfarin, NSAIDs | 6 | 18.2 |
| 2 | Febrile neutropenia / grade ≥3 neutropenia | Paclitaxel, cisplatin, methotrexate | 5 | 15.2 |
| 3 | Acute kidney injury / Cr ≥ 50 % rise | Cisplatin, aminoglycosides, ACE-Is + diuretics | 4 | 12.1 |
| 4 | Hepatocellular transaminase elevation (>5× ULN) | Methotrexate, paracetamol, azoles | 4 | 12.1 |
| 5 | Intractable nausea / grade ≥2 vomiting | Cisplatin, opioids, capecitabine | 3 | 9.1 |
| 6 | Symptomatic hypoglycaemia | Sulfonylureas + dexamethasone taper | 3 | 9.1 |
| 7 | Maculopapular rash / hypersensitivity | Penicillins, allopurinol | 3 | 9.1 |
| 8 | Sensorineural ototoxicity | Cisplatin + furosemide | 2 | 6.1 |
| 9 | Serotonin syndrome | Tramadol + SSRI | 2 | 6.1 |
| 10 | Venous thrombo-embolism | Tamoxifen, erythropoiesis-stimulating agents | 1 | 3 |
